# Dynamin-Related Protein 1 Binding Partners MiD49 and MiD51 Increased Mitochondrial Fission In Vitro and Atherosclerosis in High-Fat-Diet-Fed ApoE^-/-^ Mice

**DOI:** 10.3390/ijms25010244

**Published:** 2023-12-23

**Authors:** Jinyi Ren, Jiaqing Liu, Jiahui Zhang, Xinxin Hu, Ying Cui, Xiaoqing Wei, Yang Ma, Xia Li, Ying Zhao

**Affiliations:** 1 Molecular Medical Laboratory, College of Basic Medical Science, Dalian Medical University, Dalian 116000, China; m2634217642@163.com (J.R.); liujiaqing0302@163.com (J.L.); zhangjiahui986@163.com (J.Z.); huxinxin0816@163.com (X.H.); 13841123465@163.com (Y.C.); jcyxyyb_dy@163.com (X.W.); 17305371735@163.com (Y.M.); 2 Department of Immunology, College of Basic Medical Science, Dalian Medical University, Dalian 116000, China; 3 Liaoning Provincial Core Lab of Medical Molecular Biology, Dalian Medical University, Dalian 116000, China

**Keywords:** atherosclerosis, mitochondrial fission, MiD49, MiD51, endothelial cells

## Abstract

Novel components of the mitochondrial fission machinery, mitochondrial dynamics proteins of 49 kDa (MiD49) and 51 kDa (MiD51), have been recently described, and their potential therapeutic targets for treating cardiovascular disease have been shown, including acute myocardial infarction (AMI), anthracycline cardiomyopathy and pulmonary arterial hypertension (PAH). Here, we examined the role of MiD49 and MiD51 in atherosclerosis. MiD49/51 expression was increased in the aortic valve endothelial cells (ECs) of high-fat diet-induced atherosclerosis in ApoE^-/-^mice and IL-8-induced human umbilical vein endothelial cells (HUVECs), which accelerated dynamin-related protein 1 (Drp1)-mediated mitochondrial fission. Silencing MiD49/51 reduced atherosclerotic plaque size, increased collagen content, and decreased the IL-8-induced adhesion and proliferation of HUVECs. MiD51 upregulation resulted from decreased microRNA (miR)-107 expression and increased hypoxia-inducible factor-1a (HIF-1a) expression. Treatment with miR-107 mimics decreased atherosclerotic plaque size by reducing HIF-1α and MiD51 production. Both MiD49 and MiD51 were involved in atherosclerotic plaque formation through Drp1-mediated mitochondrial fission, and the involvement of MiD51 in this process was the result of decreased miR-107 expression and increased HIF-1α expression. The miR-107–HIF-1α–MiD51 pathway might provide new therapeutic targets for atherosclerosis.

## 1. Introduction

Atherosclerosis is a chronic and progressive vascular disease with a complex etiopathogenesis, which eventually may lead to its clinical complications, acute myocardial infarction (AMI) and stroke [1]. Endothelial barrier integrity plays a key role in maintaining fluid balance between the circulation and tissues, and vascular homeostasis. Studies indicate that endothelial dysfunction [2] and the activation of inflammatory pathways [3] are important causes of atheroma plaque formation.

Accumulating evidence demonstrates the fundamental role of mitochondrial dynamics in the pathogenesis of atherosclerosis. Mitochondria exist in dynamic networks that undergo continuous fission (division) and fusion (union) to maintain their integrity, distribution and size [4]. Altered mitochondrial dynamics in atherosclerosis result, in part, from excessive mitochondrial fission caused by increased activation of the fission-mediating GTPase dynamin-related protein 1 (Drp1) [4]. Drp1 moves from the cytosol to the outer mitochondrial membrane when activated, where it interacts with its binding partners in a multimerization reaction that culminates in fission. Novel components of the mitochondrial fission machinery, mitochondrial dynamics proteins of 49 kDa (MiD49) and 51 kDa (MiD51), have been recently described and have been shown to mediate mitochondrial fission by targeting Drp1 to the mitochondrial surface [5]. The two proteins are very similar in structure, and are both capable of endoplasmic reticulum interactions and binding to Drp1 in the same region; however, some studies have shown that as fission proteins, their deletion promotes mitochondrial elongation, and targeting just one of the proteins is sufficient to induce changes in mitochondrial morphology. The two proteins’ expression varies with age and tissue type, suggesting that there may be functional differences between the two proteins [5].

The possibility that MiD49 and MiD51 have a role in a portion of cardiovascular disease has been assessed [6,7]. MiD49 and MiD51 expression were pathologically elevated in both the media and intima of small pulmonary arteries of the lungs from humans with experimental pulmonary arterial hypertension (PAH), and silencing MiD49/51 regressed experimental PAH [6]. In anthracycline cardiomyopathy, knockdown of MiD49 was found to protect the heart against doxorubicin-induced mitochondrial fission and apoptosis in cardiotoxicity [7]. After targeting both MiD49 and MiD51 in the treatment of acute myocardial infarction, cardiomyocytes from acute ischemia–reperfusion injury were protected and the size of AMI was reduced [5]. However, whether or not MiD49/51 is also involved in the pathogenesis of atherosclerosis still needs to be clarified.

MicroRNA (miRNA) has a length of about 20–25 nucleotides and regulates post-transcriptional gene expression by specifically binding to the 3′-UTR of the target mRNAs, inhibiting protein synthesis and initiating mRNA degradation. Normally, a miRNA targets the 3′-UTR of multiple mRNAs, which indicates that a certain miRNA could activate or suppress multiple signaling pathways [8]. Recent studies have found that the progression of atherosclerosis has been linked to the dysregulation of several miRNAs, in which the expression of miR-107 in the serum of patients with atherosclerosis is lower than that in healthy controls and is involved in the functional regulation of vascular wall cells.

Here, we examined the fundamental role of MiD49 and MiD51 in the development of atherosclerosis. We reported that the expression of both MiD49 and MiD51 was pathologically elevated in the aortic valve endothelial cells (ECs) of ApoE^-/-^ mice and in IL-8-induced human umbilical vein endothelial cells (HUVECs). Immunofluorescence staining revealed that mitochondrial fission was facilitated in HUVECs induced by IL-8. Silencing MiD49 or MiD51 expression restored mitochondrial fusion and reversed IL-8-induced HUVEC proliferation and adhesion. The effects of MiD knockdown on proliferation and adhesion were due to a reduction in proliferating cell nuclear antigen (PCNA), Cyclin D1, vascular cell adhesion molecule-1 (VCAM-1) and intercellular adhesion molecule-1 (ICAM-1), and an increase in p27^Kip1^. Furthermore, the progression of atherosclerosis has been linked to the dysregulation of miR-107, which resulted in the increased expression of MiD51 by targeting hypoxia-inducible factor-1α (HIF-1α). Silencing MiD49 or MiD51 or augmenting an miR-107 mimic reversed established atherosclerosis in vivo, highlighting the contribution of the miR-107–HIF-1α–MiD51–Drp1 axis to the pathophysiology of atherosclerosis and the viability of this pathway as a target for new therapeutic strategies.

## 2. Results

### 2.1. Increased Expression of MiD49 or MiD51 in Atherosclerotic ECs

**Figure 1:**
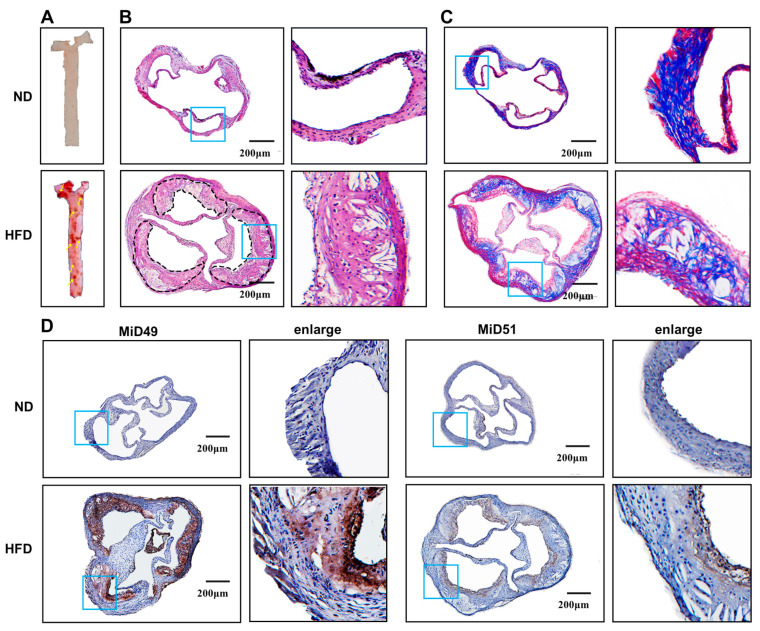

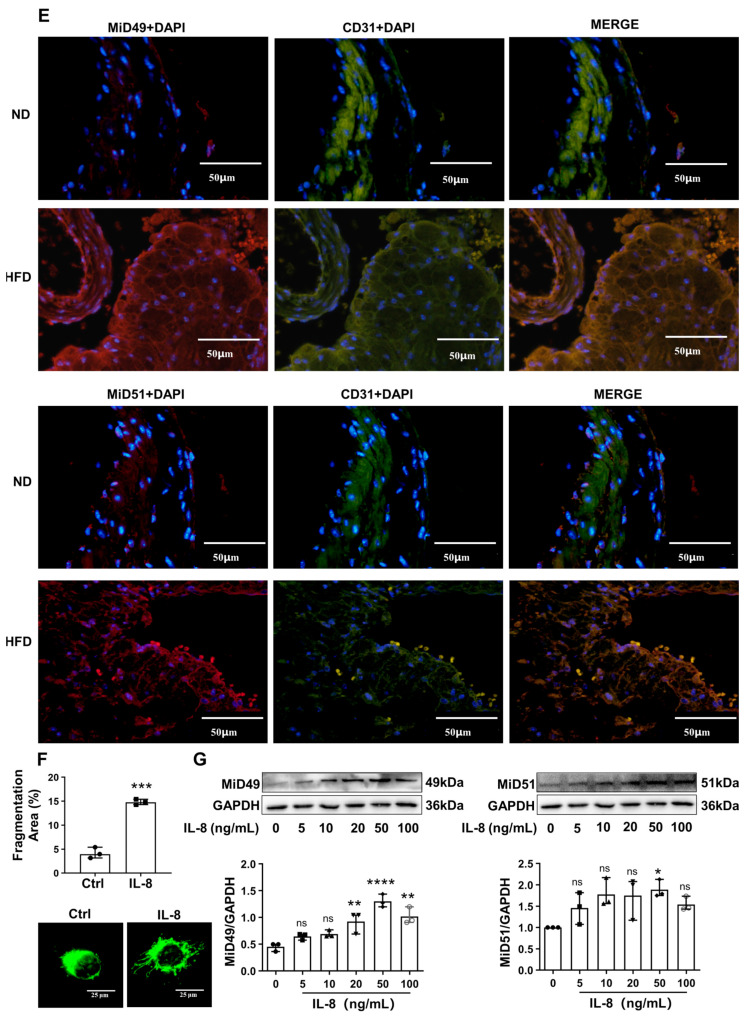
Upregulation of mitochondrial dynamics proteins of 49 and 51 kDa (MiD49 and MiD51) in HUVECs and atherosclerotic ECs. (**A**) Representative images of Oil Red O-stained aorta from the ND (normal diet) or HFD (high-fat diet) mice that were fed for 12 weeks. (**B**,**C**) HE and Masson staining performed to show the morphology and collagen of the blood vessels; atherosclerotic plaques are delineated by dashed lines (scale bars: 200 μm). (**D**,**E**) The expression of MiD49/51 in mouse aorta detected via immunohistochemistry (IHC) (scale bars: 200 μm) and immunofluorescence (IF) (scale bars: 50 μm). (**F**) Representative images of mitochondria in HUVECs stimulated with or without IL-8 for 48 h. Live cells were imaged after staining with Mitotracker (Green). Mitochondrial fragmentation was analyzed using Image J 1.53c and quantified as a percentage (*t*-test, *** *p* < 0.001; n = 3; scale bars: 25 μm). (**G**) The protein expression levels of MiD49 and MiD51 were analyzed via Western blotting (one-way ANOVA, * *p* < 0.05, ** *p* < 0.01, **** *p* < 0.0001, ns: not significant; n = 3 in each group). Data are presented as medians and ranges.

Oil Red O staining of the aorta (Figure 1A), HE staining (Figure 1B), and Masson staining (Figure 1C) of the aortic valve sections all showed that the mouse atherosclerosis model was successfully established. MiD49 and MiD51 expression were increased in the aortic valve of an atherosclerotic mouse (Figure 1D). Meanwhile, immunofluorescence staining of the aortic valve sections localized this increased expression of MiD49 and MiD51 to the endothelial cells (Figure 1E). To further understand the possible impact of MiD49 and MiD51 on atherosclerosis, the next procedure was to evaluate the role of MiD49 and MiD51 in vitro. As reported previously [3], atherosclerosis is a chronic inflammatory disease. In vitro, we used IL-8, a pro-atherogenesis inflammatory cytokine, to stimulate and observe the expression of MiD49 and MiD51 in HUVECs. In IL-8-induced HUVECs, we firstly observed that mitochondrial fission was increased compared to that in normal HUVECs (Figure 1F). Then, we also found an increased expression of MiD49 and MiD51 in HUVECs with increasing concentrations of IL-8 stimulation, and the protein levels of MiD49 and MiD51 were most significantly increased after stimulation with 50 ng/mL of IL-8 (Figure 1G).

### 2.2. Silencing MiD49 or MiD51 Inhibited Mitochondrial Fission, Cell Adhesion and Proliferation by Inhibiting Drp1 and Related Proteins

**Figure 2:**
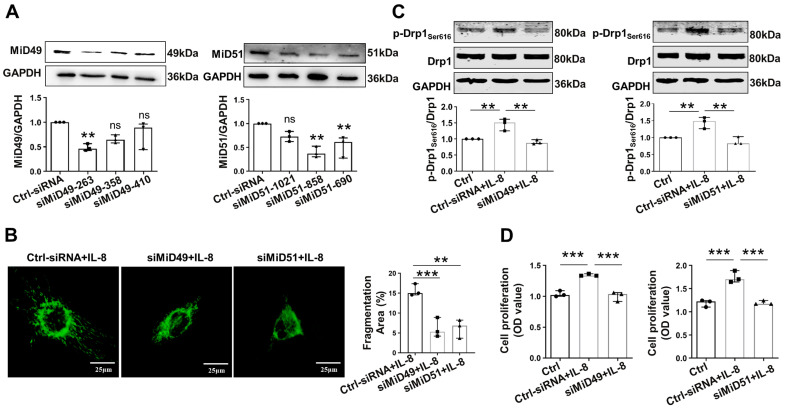

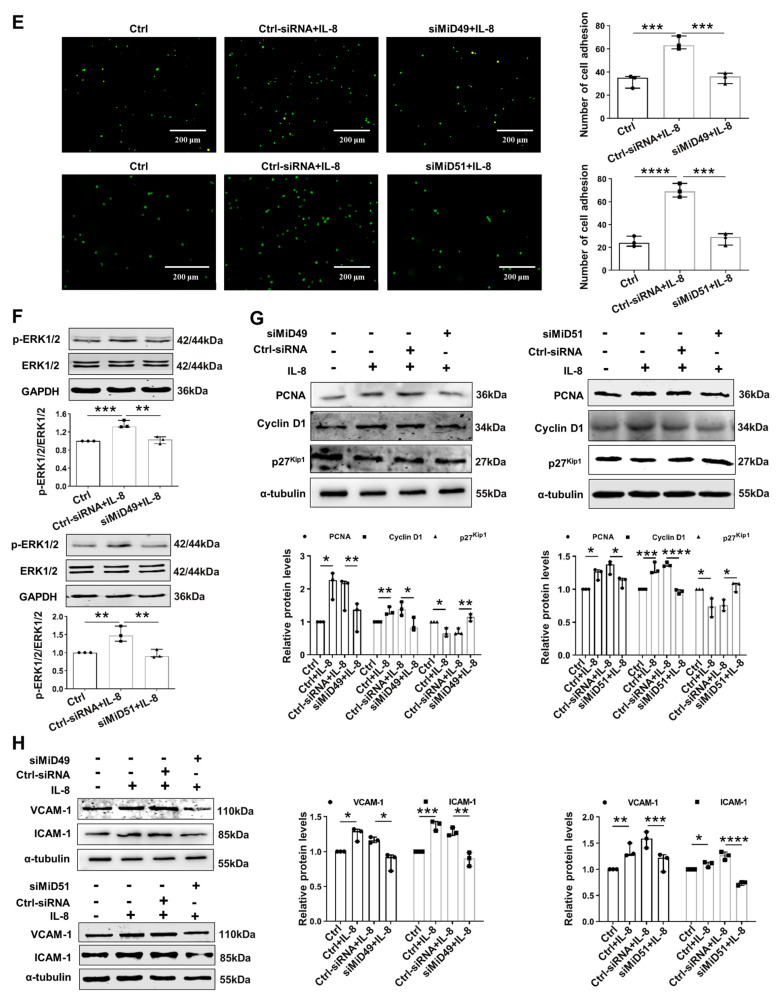
MiD49 or MiD51 regulated mitochondrial network, cell proliferation, and adhesion. (**A**) The transfection efficiencies of the three siMiD49 or MiD51 species in the HUVECs detected via Western blotting 48 h after transfection (** *p* < 0.01, ns: not significant; n = 3). (**B**) Representative images of mitochondrial network of HUVECs stained with Mitotracker (Green). Specific small interfering RNA (siRNA) was transfected into HUVECs and induced with 50 ng/mL IL-8 for 48 h before imaging, and mitochondrial fragmentation was quantified (** *p* < 0.01, and *** *p* < 0.001; scale bars: 25 μm, n = 3). (**C**,**F**–**H**) HUVECs transfected with siMiD49 or siMiD51 and induced with IL-8 at 50 ng/mL. The cells were harvested 48 h later for Western blotting. Representative images of the Western blot and the densitometries of the expressions of p-Drp1_Ser616_, Drp1, p-ERK1/2, ERK1/2, PCNA, Cyclin D1, p27^Kip1^, VCAM-1, and ICAM-1 are shown. GAPDH or α-tubulin was used as the loading control (* *p* < 0.05, ** *p* < 0.01, *** *p* < 0.001, and **** *p* < 0.0001; n = 3). Ctrl indicates control; siMiD is the siRNA against MiD49 or MiD51. (**D**) Cell proliferation analyzed 48 h after transfection by CCK8 (*** *p* < 0.001; n = 3). (**E**) Cell adhesion analyzed after the incubation of HUVECs with BCECF AM-labeled THP-1 cells for 4 h (*** *p* < 0.001 and **** *p* < 0.0001; scale bars: 200 μm; n = 3). All data are presented as medians and ranges. Statistically significant differences between groups were determined using one-way ANOVA.

We examined the impact of MiD49 and MiD51 on the functionality of HUVECs by using a specific siRNA that reduced the levels of the corresponding MiD49 and MiD51 genes in HUVECs. While transfected with three different siMiD49s and siMiD51s into HUVECs, the downregulation of MiD49 and MiD51 expression with siMiD49-263 and siMiD51-858 showed the most obvious inhibitory effects (Figure 2A). Knocking down MiDs inhibited the mitochondrial fission of IL-8-induced HUVECs (Figure 2B) and also inhibited the expression of the fission mediator p-Drp1_Ser616_ (Figure 2C). These changes actually had effects on the function of the ECs; the data showed that siMiD49 or siMiD51 significantly reduced the proliferation of HUVECs (Figure 2D) and the cellular adhesion ability of monocytes to HUVECs in IL-8 stimulation (Figure 2E).

We next investigated the possible mechanistic pathways connecting MiDs to the adhesion and proliferation of ECs. Extracellular signal-regulated kinase (ERK) promotes the phosphorylation of Drp1 at serine 616 [9,10] and increases cell proliferation [11]. The knockdown of MiD49 or MiD51 decreased ERK1/2 phosphorylation in HUVECs stimulated with IL-8 (Figure 2F). Silencing MiDs repressed the expression of proliferating cell nuclear antigen (PCNA) and Cyclin D1, and increased the expression of the cyclin-dependent kinase 4 (CDK4) inhibitor p27^Kip1^ (Figure 2G). These results suggested that the silencing of MiDs inhibited the proliferation of ECs through the regulation of important proliferation-related proteins. Moreover, silencing MiDs also downregulated the expression of intercellular adhesion molecule-1 (ICAM-1) and vascular cell adhesion molecule-1 (VCAM-1) (Figure 2H), suggesting that silencing MiDs inhibited monocyte adhesion to HUVECs in part by inhibiting the expression of ECs adhesion-related molecules.

### 2.3. Downregulation of miR-107 Mediated MiD51 Upregulation by Targeting HIF-1α in IL-8-Induced HUVECs

**Figure 3:**
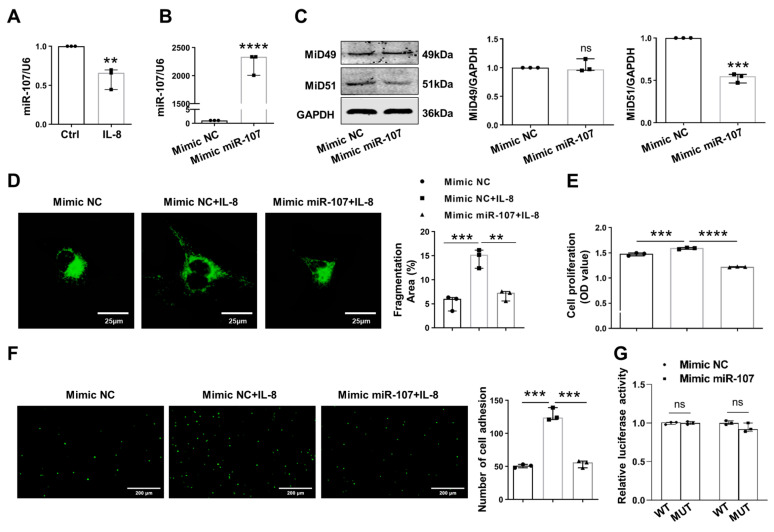

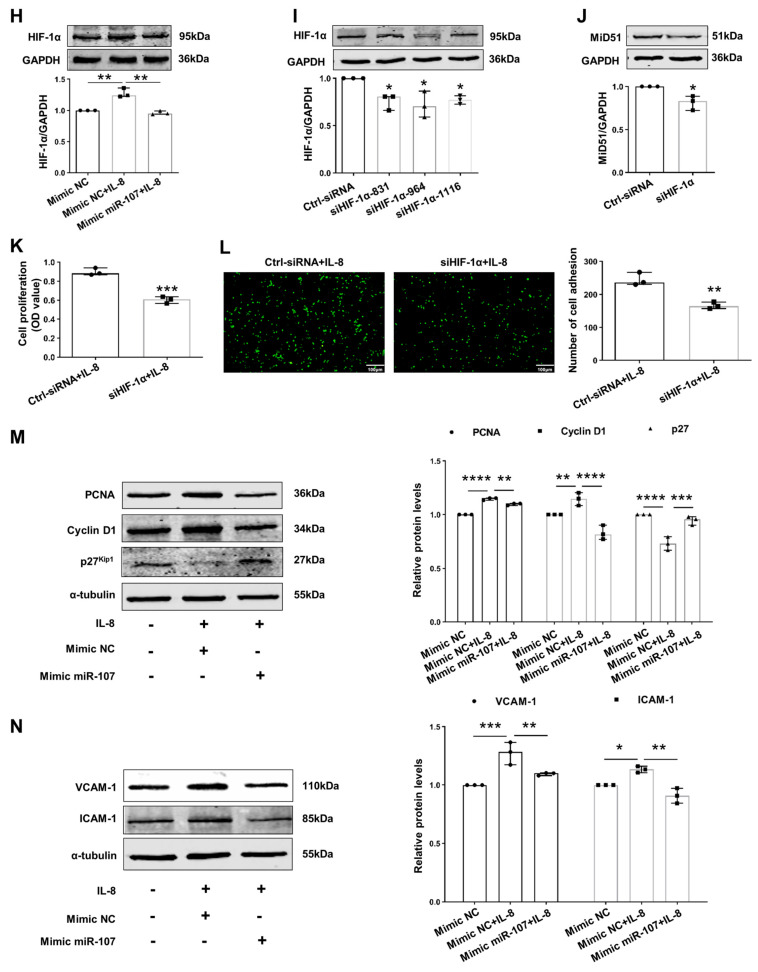
Downregulation of miR-107 mediates MiD51 upregulation by targeting HIF-1α in IL-8-induced HUVECs. (**A**,**B**) Quantification of miR-107 performed via quantitative reverse transcription–polymerase chain reaction (qRT-PCR) (*t*-test, ** *p* < 0.01, **** *p* < 0.0001; n = 3). (**C**) Representative images of Western blotting and densitometries showing the expressions of MiD49 and MiD51 in HUVECs transfected with the miR-107 mimic. Cells were transfected with miR-107 for 48 h. GAPDH was used as the loading control (*t*-test, *** *p* < 0.001, ns: not significant; n = 3). (**D**) Representative images of mitochondrial networks of HUVECs transfected with miR-107 mimic and induced with 50 ng/mL of IL-8 for 48 h (one-way ANOVA, ** *p* < 0.01 and *** *p* < 0.001; n = 3; scale bars: 25 μm). (**E**) Cell proliferation analyzed 48 h after transfection using CCK8 (one-way ANOVA; *** *p* < 0.001 and **** *p* < 0.0001; n = 3). (**F**) Cell adhesion analyzed after the incubation of HUVECs with BCECF AM-labeled THP-1 cells (one-way ANOVA, *** *p* < 0.001; scale bars: 200 μm; n = 3). (**G**) The dual luciferase-reporter was used to determine whether or not miR-107 binds to the 3′-UTR of the *MiD51* gene (WT: wild type; MUT: mutant type; two-way ANOVA, ns: not significant; n = 3). (**H**,**I**) Representative images of Western blotting and densitometries showing the expressions of HIF-1α in HUVECs transfected with the miR-107 mimic and siHIF-1α for 48 h. GAPDH was used as the loading control (one-way ANOVA, * *p* < 0.05 and ** *p* < 0.01; n = 3). (**J**) Representative images of Western blotting and densitometries showing the expressions of MiD51 in HUVECs transfected with siHIF-1α for 48 h. GAPDH was used as the loading control (*t*-test, * *p* < 0.05; n = 3). (**K**,**L**) Cell proliferation and adhesion analyzed 48 h after transfection with siHIF-1α (*t*-test, ** *p* < 0.01 and *** *p* < 0.001; n = 3, scale bars: 100 μm). (**M**,**N**) Representative images of the Western blot and the densitometries of the expressions of PCNA, Cyclin D1, p27^Kip1^, VCAM-1 and ICAM-1 in HUVECs transfected with the miR-107 mimic. α-tubulin was used as the loading control (one-way ANOVA; * *p* < 0.05, ** *p* < 0.01, *** *p* < 0.001 and **** *p* < 0.0001; n = 3). All data are presented as medians and ranges.

The progression of atherosclerosis has been linked to the dysregulation of several microRNAs (miRs), of which miR-107 exerts a protective effect on atherosclerosis [8,12]. In this study, the downregulation of miR-107 was also found in IL-8-induced HUVECs (Figure 3A). We screened potential targets for miR-107 using TargetScan 8.0 and miRPathDB 2.0 bioinformatic software and showed that MiD51 could be a potential target gene for miR-107. miR-107 mimic transfection decreased the expression of MiD51 in IL-8-stimulated HUVECs but not that of MiD49 (Figure 3B,C). Augmenting miR-107 fused the mitochondrial network, and inhibited proliferation and adhesion in IL-8-induced HUVECs (Figure 3D–F). Of note is that the binding of miR-107 to the 3′-UTR of MiD51 was not confirmed with a luciferase reporter fused to the 3′-UTR of the *MiD51* gene. The cotransfection of HEK293T cells with the miR-107 mimic and the reporter constructs did not decrease luciferase activity (Figure 3G). It has been confirmed via both a luciferase reporter and RIP assay that hypoxia-inducible factor-1α (HIF-1α) is the target gene of miR-107 because miR-107 directly inhibits the expression of HIF-1α [8,12]. Thus, we observed the role and relationship of miR-107, HIF-1α, and MiD51 in IL-8-stimulated ECs. miR-107 mimic transfection also decreased the expression of HIF-1α (Figure 3H). The knockdown of HIF-1α decreased the expression of MiD51 (Figure 3I,J) and inhibited THP-1 adhesion to HUVECs and HUVEC proliferation via IL-8 stimulation (Figure 3K,L). We also observed that miR-107 mimic transfection also decreased the expression of ICAM-1, VCAM-1, PCNA, and Cyclin D1, while increasing the expression of p27^Kip1^ in HUVECs via IL-8 stimulation (Figure 3M,N). These results suggested that the miR-107–HIF-1α–MiD51 signaling pathway was involved in the functional changes in the HUVECs induced by IL-8.

### 2.4. Potential Therapeutic Implications of MiD49 or miR-107–HIF-1α–MiD51 Pathway in the Formation of the Atherosclerotic Plaque of Mice

**Figure 4:**
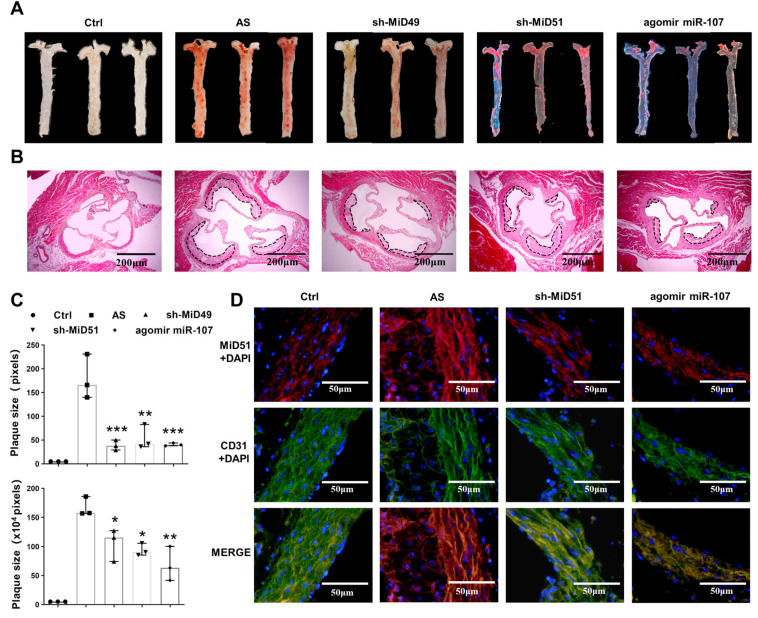

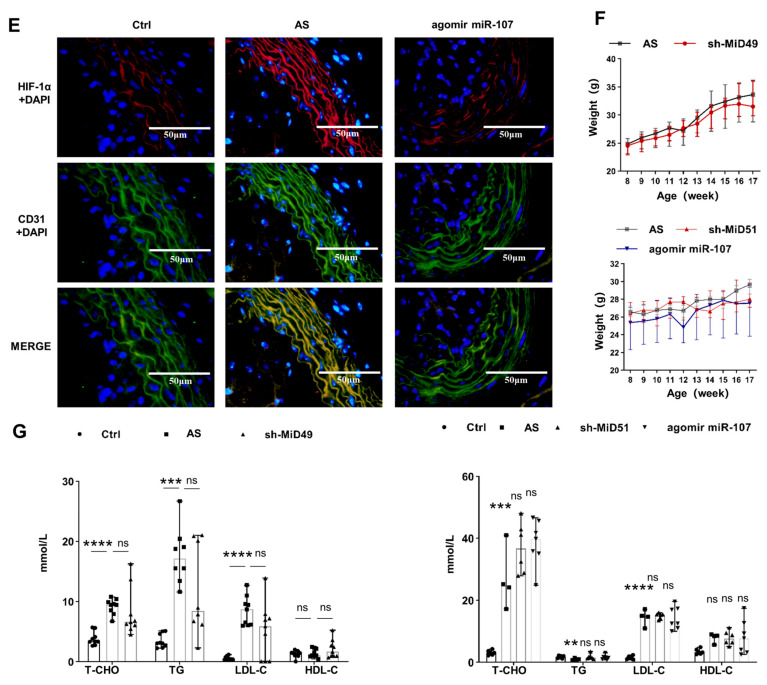
Therapeutic implications of MiD49 or the miR-107–MiD51–HIF-1a pathway in the formation of atherosclerotic plaque in mice. (**A**) Oil Red O staining performed to show the lipid deposits of the blood vessels (n = 3). Representative images derived from normal-fed C57 mice and high-fat-diet-fed ApoE^-/-^ mice that were administered sh-MiD49, sh-MiD51 or agomir miR-107 (miR-107 mimic) are shown. (**B**) Representative images of HE stained sections. Atherosclerotic plaques are delineated by dashed lines. Scale bars: 200 µm. (**C**) Statistical analysis data of Oil Red O staining (up) and HE staining (down) (one-way ANOVA, * *p* < 0.05, ** *p* < 0.01, *** *p* < 0.001; n = 3). (**D**,**E**) The expression of MiD51 and HIF-1α in mouse aortic ECs (CD31) detected using IF (blue, DAPI; red, MiD51 or HIF-1α; green, CD31; n = 3, scale bars: 50 μm). (**F**) Changes in body weight of mice during treatment (*t*-test or one-way ANOVA; n ≥ 4). (**G**) Graph showing that mice were sacrificed and blood samples were taken to detect lipid levels (one-way ANOVA; ** *p* < 0.01, *** *p* < 0.001 and **** *p* < 0.001, ns: not significant; n ≥ 4). All data are presented as medians and ranges.

The therapeutic efficacy of silencing MiD49/51 or augmenting miR-107 was determined by injecting the tail vein of mice early in the course of atherosclerosis with sh-RNA targeting MiD49/51 or a miR-107 mimic. Treatment with either siMiDs or the agomir miR-107 markedly decreased the formation of plaque in mouse aorta (Figure 4A–C). The miR-107 mimic significantly reduced the expression of MiD51 and HIF-1a on the ECs of mouse aorta (Figure 4D,E). Interestingly, none of the treatments had effects on serum lipid levels or body weight in the mice (Figure 4F,G).

## 3. Discussion

This study clarified the role of the Drp1 binding partners MiD49 and MiD51 in the pathogenesis of atherosclerosis and has five novel findings: (1) MiD expression was increased in the vascular ECs of atherosclerotic mice. (2) Inhibiting MiD expression prevented atherosclerosis by reducing mitochondrial fission. (3) The MiD inhibitor reduced EC proliferation and adhesion. (4) MiD51 expression was upregulated by miR-107 targeting HIF-1α. (5) These discoveries were translated into two new therapies that attenuate atherosclerosis; that is, the progression of atherosclerotic plaque formation might be retarded by inhibiting the miR-107–HIF-1α–MiD51–Drp1 or MiD49–Drp1 pathway.

Intimal proliferative lesions are important features of atherosclerosis [13]. It is now clear that mitochondrial fusion and fission in mitochondrial dynamics are very important for mitochondrial homeostasis [14]. Excessive mitochondrial fission may be detrimental as it causes cell proliferation, cellular metabolic disorders, cell migration, etc. In an ex vivo aortic ring assay, Drp1 inhibition significantly reduced vascular neointima formation and vascular smooth muscle cell proliferation and migration in a model of rat carotid artery balloon injury [4,15]. Drp1 inhibition was also found to reduce endothelial dysfunction and atherosclerosis in ApoE^-/-^ diabetic mice [4,16]. Our study clearly showed that the Drp1 binding partners MiD49 and MiD51 were highly expressed in the endothelial cells of the atherosclerotic vascular wall. Changes in the cellular environment, especially in the pathological state, can trigger changes in the function or activity of mitochondrial fusion- or fission-related proteins, and changes in fusion- and fission-related proteins directly affect the process of mitochondrial fusion and fission. Atherosclerosis is also a chronic inflammatory disease [17]. We found that in the vascular endothelial cells stimulated by IL-8, the upregulation of MiD49 and MiD51 increased mitochondrial fission and activated phosphorylated Drp1, leading to pathological mitochondrial fragmentation. Furthermore, the downregulation of either MiD49 or MiD51 restored mitochondrial fusion and the levels of p-Drp1, which is consistent with the role of Drp1 in atherosclerosis reported by others [15,16]. Moreover, our observations are further supported by the fact that the expression and function of the MiDs are critical determinants of mitochondrial dynamics.

Monocyte adhesion to the endothelium and endothelial cell proliferation are very important causes of intimal proliferative lesions in atherosclerosis. We found that silencing MiD49 or MiD51 reduced HUVEC proliferation and adhesion upon IL-8 stimulation. We also identified the mechanism by which MiDs regulate fission and cell function. Drp1 phosphorylation can be initiated by ERK [9,10], which activates Drp1 through the phosphorylation of serine 616 [18]. We found that silencing MiDs inhibited ERK1/2 and decreased the phosphorylation of Drp1 at serine 616, supporting the dual mechanism of MiD-induced fission. Cyclin D1, namely G1/S-specific cyclin-D1, promotes cell proliferation mainly by binding and activating cyclin-dependent kinase (CDK) 4, which is specific to the G1 phase [19]. PCNA is a kind of protein that only exists in the stage of cell proliferation and helps cells in the S phase to synthesize DNA. Our results showed that silencing MiDs could inhibit the proliferation of endothelial cells by inhibiting PCNA and Cyclin D1 and promoting p27^Kip1^. Endothelial cells change from anti-adhesion cells to cells promoting adhesion, attracting monocytes to adhere to the vascular wall and to infiltrate it, which is one of the important causes for atherosclerosis. ICAM-1 and VCAM-1, which are highly expressed in atherosclerotic endothelial cells, are important adhesion molecules that mediate adhesion reactions [20]. Our study showed that silencing MiD51 in IL-8-induced HUVECs could reduce ICAM-1 and VCAM-1 expression and subsequently inhibit the adhesion of THP-1 to HUVECs. Therefore, the siMiD-mediated inhibition of ECs proliferation and adhesion could be explained by the fact that silencing MiDs inhibits the activity of ERK1/2 and Drp1, as well as the expression of PCNA, Cyclin D1, ICAM-1, and VCAM-1, and activates p27^Kip1^, resulting in decreased CDK4 activity. Taken together, these results showed a multifactorial mechanism underlying the inhibition of mitochondrial fission and EC proliferation and adhesion, achieved by silencing MiDs. Meanwhile, these results also showed the consistency of the roles of MiD49 and MiD51 in these processes.

MiD49 and MiD51 proteins are very similar in structure; both are capable of endoplasmic reticulum interaction and bind Drp1 in the same region; thus, as we and others [5,6] show in the results, the two proteins exhibit the same role in some diseases. Nevertheless, the present study identified depressed miR-107 expression as the primary driver of pathological MiD51 upregulation in atherosclerosis, but miR-107 had no effect on MiD49, which suggested the possible differences in the regulatory pathways of the two proteins. Our study showed that the ability to decrease MiD51 through the over-expression of miR-107 confirms the central role played by MiD51 in the mitochondrial fission phenotype and EC proliferation and adhesion in atherosclerosis. Although bioinformatics showed that MiD51 had a potential binding site with miR-107, unfortunately, this prediction was not confirmed by the direct experimental evidence of the dual-luciferase reporter. HIF-1α is an important transcriptional factor associated with angiogenesis, aerobic glycolysis, and pro-inflammatory processes, which are also confirmed to be risk factors for atherosclerosis [21,22]. HIF-1α activation and subsequently Drp1_Ser616_ phosphorylation are involved in mitochondrial fission; however, these findings were derived from cancer cells or the pancreas [23,24,25,26].

## 4. Materials and Methods

### 4.1. Animal Models and Treatment

Homozygous male ApoE^-/-^ mice on C57/BL background and wild-type C57/BL mice at 8 weeks were purchased from Changsheng Biotechnology Co., Ltd. (Shenyang, China). All mice were housed in the Dalian Medical University SPF, a temperature-controlled, pathogen-free environment, in a 12 h light/dark cycle with ad libitum access to food and water. All ApoE^-/-^ mice were randomized into five groups as follows: (1) high-fat diet (HFD); (2) HFD + sh-MiD49; (3) HFD + sh-MiD51; (4) HFD + agomir miR-107. The high-fat diet (XT108C) was purchased from Xietong Shengwu (Nanjing, China). The composition of the high-fat diet includes 22.62% protein, 45.51% carbohydrate, 20.06% fat and 1.25% cholesterol. Six wild-type C57/BL male mice served as controls and were fed a regular diet. From the 10th week, sh-MiD51 or sh-Mid49 (GenePharma, Suzhou, China) in an amount of 100 μL (titer 10^9^) was injected once. Agomir miR-107 (GenePharma, Suzhou, China) was administered at 0.8 nmol/g once a week 8 times. All mice were euthanized via cervical dislocation under anesthesia after 18 weeks. The animal protocol was approved by the local research ethics review board of the Animal Ethics Committee of Dalian Medical University and was in conformity with the Guide for the Care and Use of Laboratory Animals published by the U.S. National Institutes of Health (8th edition, National Academies Press).

### 4.2. Reagents and Antibodies

The antibodies against MiD51, Drp1, p-Drp1, ERK1/2, p-ERK1/2, p27^Kip1^, Cyclin D1, VCAM-1, ICAM-1, HIF-1α and CD31 were purchased from Proteintech (Wuhan, China). Bovine Serum Albumin (BSA) and 4, 6-diamidino-2-phenylindole (DAPI) were obtained from Abcam Inc. (Cambridgeshire, UK). Synthetic oligonucleotide siMiD51-690, siMiD51-858, siMiD51-1021 and miR-107 mimics, and negative controls were purchased from Genepharma (Suzhou, China). Goat anti-Rabbit IgG (H + L) and anti-Mouse IgG (H + L) secondary antibodies were purchased from Thermo Scientific (Waltham, MA, USA). A total cholesterol assay kit, triglyceride assay kit, high-density lipoprotein cholesterol assay kit and low-density lipoprotein cholesterol assay kit were purchased from Jiancheng Bioengineering Institute (Nanjing, China).

### 4.3. Cell Culture

Human umbilical vein endothelial cells (HUVECs) were purchased from the ATCC (American Type Culture Collection, Manassas, VA, USA) and maintained in Dulbecco’s modified Eagle’s medium (DMEM; Procell, Wuhan, China) containing 10% fetal bovine serum (FBS; Excell Bio, Taicang, China) and 1% penicillin/streptomycin. CBCAS (Cell Bank of the Chinese Academy of Sciences, Shanghai, China) provided human monocyte THP-1 cells. To prepare buffered RPMI-1640, a packet of RPMI-1640 powder (Gibco, Waltham, MA, USA) was mixed with 3.7 g of NaHCO_3_ (Tianhe Chemical Reagent Factory, Tianjin, China) in 1 L of water and filtered through a sterile filter. We cultured the cell lines in 5% CO_2_ at 37 °C.

### 4.4. Immunohistochemistry

Sections of the heart’s aorta root were dewaxed and hydrated with xylene and gradient ethanol. The sections were placed in citric acid buffer at 94 °C for 15 min to repair the antigen. An appropriate amount of endogenous peroxidase blocker was added dropwise to the slides for 10 min at room temperature. Subsequently, the sections were blocked with 5% BSA at room temperature for 30 min and incubated with the corresponding primary antibodies in a humidified chamber overnight. A universal two-step detection kit was used for the secondary processing of tissue samples in accordance with the manufacturer’s instructions (Zhongshan Golden Bridge Biotechnology Co., Ltd., Beijing, China). A DAB stain and hematoxylin counterstain were used. The sections were dehydrated in gradient ethanol, transparentized with xylene and finally sealed with neutral gel.

### 4.5. Immunofluorescence Staining

The components of the immunofluorescence detection manipulation method were described in the immunohistochemistry section. After antigen repair with citrate, the cells were blocked in PBS with 5% BSA at room temperature for 30 min. Briefly, slides were incubated with primary antibodies in a 37 °C wet box for 1 h. Then, they were washed in PBS and incubated with fluorescent-labeled secondary antibody for 40 min. Finally, each sample was sealed with tablets containing DAPI, which was used to stain cellular nuclei, and the images were scanned under fluorescence microscopy.

### 4.6. Cell Transfections

HUVEC cells were inoculated into 6-well plates (1.5 × 10^5^ cells/well) and cultured for 24 h, and transfection was not performed until 80–90% confluence was reached and growth was in good condition. The siRNA or miRNA mimic (GenePharma, Suzhou, China) was added to the media at a final concentration of 50 nM. In accordance with the instructions of the Lipofectamine^TM^2000 (Invitrogen, Waltham, MA, USA), transfection was conducted.

siMiD51-690 sense: 5′-GGAUGUACGAUCGGGCGAUTT-3′
siMiD51-690 antisense: 5′-AUCGCCCGAUCGUACAUCCTT-3′
siMiD51-858 sense: 5′-CCUUCGACACAGAUACAUUTT-3′
siMiD51-858 antisense: 5′-AAUGUAUCUGUGUCGAAGGTT-3′
siMiD51-1021 sense: 5′-GCAAGCUGCUGUGGACAUATT-3′
siMiD51-1021 antisense: 5′-UAUGUCCACAGCAGCUUGCTT-3′
negative control sense: 5′-UUCUCCGAACGUGUCACGUTT-3′
negative control antisense: 5′-ACGUGACACGUUCGGAGAATT-3′
hsa-mir-107 Mimics sense: 5′-AGCAGCAUUGUACAGGGCUAUCA-3′
hsa-mir-107 Mimics antisense: 5′-AUAGCCCUGUACAAUGCUGCUUU-3′
negative control sense: 5′-UUCUCCGAACGUGUCAGGUTT-3′
negative control antisense: 5′-ACGUGACACGUUCGGAGAATT-3′

### 4.7. Cell Proliferation Assay

Cell proliferation was assessed by using the CCK8-based assay. The transfected cells were plated in 96-well microplates at a density of 4 × 10^3^ cells per well. After the cells adhered to the walls, 50 ng/mL of IL-8 was added to a portion of the wells, and the rest were left untreated as a control, according to the different groups. After incubation for 48 h, the original medium was removed and replaced with fresh medium containing 10% CCK8. The reaction was protected from light in an incubator for 1–4 h, and then the absorbance was read at 450 nm on a microplate reader.

### 4.8. Cell Adhesion Assay

THP-1 cells with good growth status were harvested. After centrifugation, the cells were resuspended with a 10 μM fluorescent probe of BCECF AM (Beyotime, Shanghai, China) in 1640 medium and incubated in the incubator for 1 h. After re-centrifugation, cells were washed 2–3 times with 1640 medium, resuspended in 1640 medium with 10% FBS and prepared as suspension THP-1 cells. After transfection, the experimental group (IL-8) and control group (equivalent volumes of medium) were set up. After incubation for 48 h, the media were removed before THP-1 cell suspension. After 4 h of incubation while shielded from light, cells were washed 2–3 times with PBS. Photographs were taken under a fluorescence microscope.

### 4.9. miR-Binding Luciferase Reporter Assay

To validate the binding of candidate miR-107 to MiD51 mRNA, binding assays were performed using the 3′-UTR of MiD51 and miR-107. The reporter plasmid containing the 3′-UTR of MiD51 was derived from Genepharma. Briefly, 293T cells were cotransfected with MiD51 reporter plasmids together with miR-control or miR-107 with GP-transfect-Mate. The luciferase activity was detected using a multifunctional microplate reader.

### 4.10. Quantification of Atherosclerotic Lesion Area

The lipid area and plaque size in the image were measured using Image J 1.53c.

### 4.11. Quantification of Mitochondrial Fragmentation

Live cells were imaged after staining with Mitotracker Green (10 nmol/L, 30 min in culture medium at 37 °C; Beyotime, shanghai, China). The cells were imaged using an Olympus microscope. Image J was used to threshold the obtained images, set the unit as a pixel and set measurements (check area and limit to threshold) to obtain the total mitochondrial area and fragmentation area.

### 4.12. Statistical Analyses

All research results were analyzed using GraphPad Prism 9 and presented as medians and ranges. Student’s *t*-test was used to analyze statistically significant differences between two groups. ANOVA was used to estimate the significant differences between the values of at least 3 groups. A value of *p* < 0.05 was considered to indicate a statistically significant difference.

## 5. Conclusions

**Figure 5:**
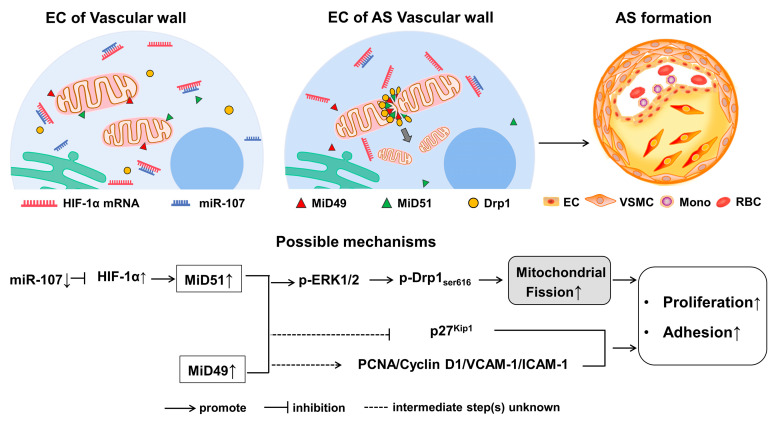
Schematic representation of the relevance between atherosclerosis and the mitochondrial dynamic protein (MiD) pathway.

Our in vitro data demonstrated that inhibiting either MiD49 or MiD51 caused a sustained state of mitochondrial fusion, which decreased EC proliferation and adhesion, and that the inhibition of MiD51 could be achieved through miR-107 targeting HIF-1α. These findings show the biological plausibility of the therapeutic targeting of MiD49 and MiD51 or the augmentation of miR-107 in atherosclerosis. In vivo, we confirmed these therapeutic benefits by injecting mice’s tail veins with either silenced MiD49/51 or augmented miR-107. The siMiD49, siMiD51 and miR-107 mimic regressed experimental atherosclerosis, as did the treatment with the miR-107 mimic, which reduced the expression of MiD51 and HIF-1α proteins. This study further implicated the dysregulation of mitochondrial dynamics as a potential therapeutic target in atherosclerosis (Figure 5).

## Data Availability

The data sets generated and/or analyzed during the current study are available from the corresponding author upon reasonable request.
